# Novel Feature in patient with Complex PTSD in Syria: Case Report

**DOI:** 10.1192/j.eurpsy.2023.2073

**Published:** 2023-07-19

**Authors:** A. Alhaj

**Affiliations:** Psychiatry, Alasadi Hospital, Damascus, Syrian Arab Republic

## Abstract

**Introduction:**

Complex PTSD is a new trauma-related disorder in ICD 11 and it differs from PTSD by the trauma being repetitive or prolonged and some other symptoms, while selective anosmia is the inability to detect a particular odor.

**Objectives:**

Is there any relationship between severe trauma and selective anosmia as a novel feature?

**Methods:**

INTERNATIONAL TRAUMA QUESTIONNAIRE (ITQ), Case report and non-systematic review through literature research in PubMed database, using the key-word “selective anosmia”

**Results:**

Two articles suggest that selective anosmia may present in animals when olfactory epithelium exposures to some chemical substances while the other two articles indicate that cortical nucleus in the amygdala has major olfactory connections and its degeneration is likely to contribute to the early selective anosmia common in Parkinson’s disease, but no one described this feature in psychiatric trauma.

**Conclusions:**

May be this is the first time ever to describe selective anosmia during severe trauma suggesting a role of amygdala as in this case report, therefore Syria and other countries that had similar crisis need more studies to get accurate statistics, explore more rare features, and test the effectiveness of treatment options.

**Disclosure of Interest:**

None Declared

